# Discovery of Isobavachin, a Natural Flavonoid, as an Apolipoprotein E4 (ApoE4) Structure Corrector for Alzheimer’s Disease

**DOI:** 10.3390/molecules30040940

**Published:** 2025-02-18

**Authors:** Sachin P. Patil, Bella R. Kuehn, Christina McCullough, Dean Bates, Hadil Hazim, Mamadou Diallo, Naomie Francois

**Affiliations:** 1 NanoBio Laboratory, Widener University, Chester, PA 19013, USA; 2 Department of Chemical Engineering, Widener University, Chester, PA 19013, USA

**Keywords:** Alzheimer’s disease, Apolipoprotein E4 (ApoE4), flavonoids, Isobavachin, structure correctors, protein stabilization, bioavailable compounds, molecular docking, molecular dynamics

## Abstract

Alzheimer’s disease (AD) is a progressive neurodegenerative disease characterized by extensive neurodegeneration and consequent severe memory loss. Apolipoprotein E4 (ApoE4) is the strongest genetic risk factor for AD, with its pathological effects linked to structural instability and altered interactions with lipids and other important disease proteins including amyloid beta (Aβ) and tau (*τ*). Therefore, correcting and stabilizing the ApoE4 structure has emerged as a promising therapeutic strategy for mitigating its detrimental effects. In this study, we investigated naturally occurring bioavailable flavonoids as ApoE4 stabilizers, focusing on their potential to modulate ApoE4 structure and function. A comprehensive investigation of a focused database using our integrated computational and experimental screening protocol led to the identification of Isobavachin as a potential corrector and stabilizer of ApoE4 structure. In addition, a few other bioavailable flavonoids with similar stabilizing properties were identified, albeit to a much lesser extent as compared to Isobavachin. The findings support the therapeutic potential of flavonoids as ApoE4 modulators and highlight Isobavachin as a lead candidate for further preclinical evaluation. These results provide new insights into the pharmacological targeting of ApoE4 and open avenues for the development of flavonoid-based, ApoE-directed therapies for AD.

## 1. Introduction

Alzheimer’s disease (AD) is a progressive neurodegenerative disorder and the most common form of dementia, posing an immense socioeconomic challenge, with over 55 million people living with AD and other dementia worldwide [1]. As per the Global Burden of Disease (GBD) database (accessed on 5 February 2025), the disability-adjusted life-years (DALYs) for AD and other dementias have more than doubled from 2000 to 2021 (2000: 14,553; 2021: 33,627). Clinically, AD is characterized by significant memory loss, cognitive decline, and eventual loss of functional independence [2]. Pathologically, it involves the accumulation of extracellular amyloid-beta (Aβ) plaques and intracellular neurofibrillary tangles composed of hyperphosphorylated tau (*τ*) protein [3,4,5,6].

Numerous studies imply the precedence of Aβ accumulation before other AD-related phenotypes, including neurofibrillary *τ* tangle formation, reactive gliosis, activated microglia and complement pathways, severe inflammation, early synapse loss, and neurotransmitter deficit, leading in turn to irreversible cognitive impairment [7,8,9]. Accordingly, the “amyloid cascade hypothesis” has been considered central to the AD pathogenesis, receiving major preclinical and clinical research attention so far [10]. In this context, two anti-Aβ monoclonal antibodies, viz., Aducanumab and Lecanemab, have recently received accelerated approvals by the United States Food and Drug Administration (FDA) [11,12]. Despite their effectiveness in reducing brain Aβ accumulation, the therapeutic efficacy of these approved and other investigational Aβ-directed approaches on cognitive decline is unfortunately somewhat limited [13]. Moreover, serious adverse effects, including brain edema, together with high costs, plague the widespread use of these biological drugs against AD. As such, the European Medical Agency (EMA) declined to license Aducanumab across Europe, citing a lack of safety and clinical efficacy, and the manufacturer Biogen abandoned its commercial development completely in January 2024. Thus, despite decades of research, the therapeutic landscape for AD remains limited. Indeed, the current pharmacological approaches for AD include three cholinesterase inhibitors (Donepezil, Galantamine, and Rivastigmine) and a N-methyl-d-aspartate (NMDA) receptor antagonist (Memantine), all of which have beneficial but only short-lived (lasting ~6 months) effects in mediating only the symptoms of AD [13]. Therefore, there is a significant unmet need for novel, effective therapeutic approaches targeting alternative, fundamentally important disease pathways in AD.

Among such pathways, the role of Apolipoprotein E (ApoE), particularly its isoform-specific effect, is recently receiving significant traction as a promising avenue for AD intervention. ApoE, a 299-amino acid glycoprotein, is the primary lipid transporter in the brain, which exists in three major isoforms, viz., ApoE2, ApoE3, and ApoE4, differing by single amino acid substitutions at positions 112 and 158 [14]. These minor structural differences differentially impact ApoE risk for AD, with ApoE4 identified as the strongest genetic risk factor for late-onset AD, conferring a 10–15-fold increased risk in homozygous carriers compared to neutral ApoE3 [14]. The structural instability of ApoE4 profoundly impacts its biochemical properties, including lipidation, receptor binding, protein stability, and aggregation propensity, exacerbating various neurodegenerative processes in AD [15,16,17]. Specifically, poorly lapidated ApoE4 impairs cellular lipid homeostasis, leading to exacerbation of Aβ deposition [18,19]. Also, ApoE4 binds less efficiently to its receptors including low-density lipoprotein receptors (LDLRs) and heparan sulfate proteoglycans (HSPGs), impairing lipid and Aβ clearance [20]. Furthermore, ApoE4 is less stable than ApoE3, leading to an increased tendency for self-aggregation, further contributing to its neuronal toxicity. Thus, stabilizing ApoE4 structure to prevent its pathological conformational changes has emerged as a promising therapeutic strategy against AD [21].

One of the well-studied approaches to stabilize ApoE4 structure is through targeting its “domain interaction”—a salt bridge interaction between the amino-terminal LDLR binding domain and the carboxy-terminal lipid binding domain [22]. This is directly attributed to the presence of Arginine at amino acid position 112 in ApoE4, instead of Cysteine112 observed in ApoE3, leading to the salt bridge interaction between Arginine61 in amino-terminal domain and Glutamate255 in carboxyl-terminal domain in ApoE4. This domain interaction in ApoE4 is found to lead to its closed structure as compared to more open structures of both ApoE2 and ApoE3 [23]. These ApoE structural differences, owing to the presence or absence of domain interaction, are responsible for their functional and related stability difference (ApoE2 > ApoE3 > ApoE4). Several small-molecule structure correctors that prohibit this domain interaction and convert ApoE4 to an ApoE3-like, more stable structure have been reported to mitigate the neurotoxic effects of ApoE4 [23,24].

**Figure 1:**
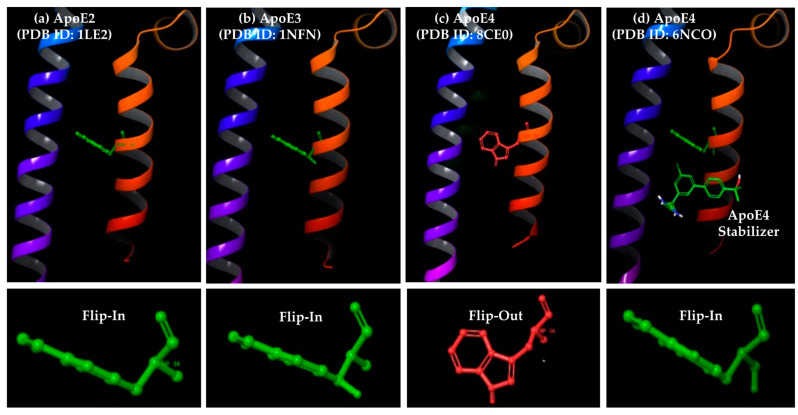
Crystal structures of different ApoE isoforms and orientation of Tryptophan34 residue. (**a**–**c**) Ribbon representation of ApoE2/E3/E4 apo structures, with Tryptophan34 shown as balls-and-sticks model; (**d**) the ApoE4 protein in complex with a small-molecule stabilizer from AbbVie Pharmaceuticals. The structures of ApoE4 stabilizers from AbbVie are shown in Supporting Figure S1.

More recently, an alternative approach in terms of stabilizing ApoE4 structure was reported by AbbVie Pharmaceuticals research team, involving small molecules that bind to an allosteric region of ApoE4 away from the domain interaction site [25]. Specifically, the Cysteine-to-Arginine substitution at 112 position was observed to induce long-distance conformational changes in ApoE4 protein. One such prominent change involves Tryptophan34 amino acid side chain assuming a “flip-out” orientation parallel to the ApoE4 axis as opposed to a “flip-in” orientation perpendicular to the axis of both ApoE2 and ApoE3 proteins (Figure 1). The small molecules identified by AbbVie team bound to a small but well-defined cavity formed by the rotation of the Tryptophan34 sidechain in ApoE4, stabilizing its structure and consequently suppressing inflammation in the neuronal cell culture [25]. Importantly, as seen in the crystal structure of ApoE4 in complex with the stabilizer molecule (Figure 1d), the ligand binding in the ApoE4 pocket is shown to shift the orientation of the Tryptophan34 sidechain to that observed in both the free ApoE2 and ApoE3 structures (Figure 1a,b). Taken together, small-molecule correctors that can interact with Tryptophan34 residue in ApoE4 and induce its proper sidechain orientation may help in modifying the aberrant conformation of ApoE4 and in turn abolish its neurotoxic and other AD-relevant downstream effects.

Therefore, given the critical role of ApoE4 in AD pathology, our present study focused on identifying small molecules that can stabilize the ApoE4 structure, thus reducing its aggregation propensity and restoring its function to resemble ApoE2/E3. Here, we utilized an integrated computational and experimental screening approach to systematically screen a focused database of bioactive small molecules for potential ApoE4 stabilizers. Specifically, the ensemble rigid-receptor docking approach was first used against two of each apo- and holo-ApoE4 crystal structures. This structure-based approach combined with our innovative consensus scoring analysis significantly enhanced the ranking for the known ApoE4 stabilizers and further led to the identification of several novel compounds with favorable molecular characteristics. These molecules were then subjected to flexible receptor docking and molecular dynamics studies, revealing Isobavachin, a bioavailable flavonoid, as a top virtual hit with the potential to bind to ApoE4 pocket and change the trytophan34 sidechain orientation towards resembling ApoE2/E3. The biophysical binding studies using surface plasmon resonance (SPR) confirmed potent Isobavachin–ApoE4 binding with nanomolar range potency. Furthermore, we highlight a few additional flavonoids with similar but relatively weaker ApoE4 stabilizing potential.

In summary, our present work provides critical insights into the pharmacological targeting of ApoE4 and lays the groundwork for developing flavonoid-based therapies for AD. Isobavachin and other active flavonoids may serve as guiding points for the discovery and optimization of more potent, selective small-molecule stabilizers for ApoE4, one of the most promising therapeutic targets against AD. Importantly, the present results also validate potential viability of the combined computational and experimental screening protocol presented here for screening other available databases for identification of potentially novel ApoE4 stabilizers. Such molecules, by targeting ApoE4’s structural instability, may hold promise as disease-modifying therapies for AD.

## 2. Results

The present study involved the use of combined structure- and ligand-based screening of a database containing drug-like compounds derived from Traditional Chinese Medicine (TCM), viz., Database of Constituents Absorbed into the Blood and Metabolites of TCM (DCABM-TCM) [26]. Natural compounds are a precious source of novel chemical scaffolds and have been shown to be basis of three-fourths of drugs approved during the past half century worldwide [27]. As such, the DCABM-TCM database may prove to be a great resource of active natural products for AD drug development [28,29], offering potential ApoE4 stabilizers that are both bioactive and bioavailable. Therefore, virtual screening hits from the DCABM-TCM database were subjected to further computational analysis, including induced-fit docking and molecular dynamics simulations followed by experimental ApoE4 binding studies using SPR.

### 2.1. Selection of the ApoE Structures for Virtual Screening

**Figure 2:**
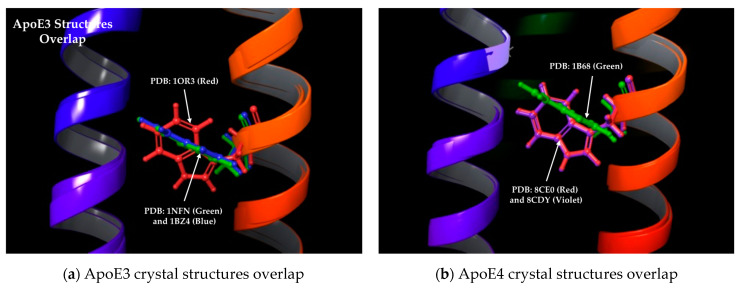
Comparison of ApoE3 and ApoE4 structures: (**a**) two ApoE3 structures (PDB IDs: 1NFN, 1BZ4) have Tryptophan34 sidechain in expected “flip-in” orientation (green and blue), while one structure (PDB ID: 1OR3) has it in “flip-out” position (red) resembling ApoE4; (**b**) two ApoE4 structures (PDB IDs: 8CE0, 8CDY) with Tryptophan34 sidechain in expected “flip-out” position (red and violet), with one structure (PDB ID: 1B68) in “flip-in” orientation (green) similar to ApoE3.

All three isoforms of ApoE are widely studied, with numerous 3D structures published using various methods, such as X-ray crystallography, Nuclear Magnetic Resonance (NMR) and Electron Microscopy (EM). Specifically, the UniProt database lists 22 X-ray structures, 5 NMR structures, and 1 EM structure for various ApoE isoforms (UniProt ID: P02649). For the present study, we focused on ApoE structures with a high resolution of ≤2 Å, leading to a total of 17 structures. Furthermore, we shortlisted only natural, non-mutant structures, resulting in the final selection of three ApoE3 structures (PDB IDs: 1NFN, 1BZ4, and 1OR3), three ApoE4 structures (PDB IDs: 1B68, 8CE0, and 8CDY) and two holo-ApoE4 structures bound with small-molecule stabilizers (PDB IDs: 6NCN and 6NCO). The careful inspection of these eight ApoE crystal structures showed that one ApoE3 structure (PDB ID: 1OR3) showed a Tryptophan34 side chain, assuming a “flip-out” orientation that resembled the ApoE4 structure, while one ApoE4 structure (PDB ID: 1B68) with a Tryptophan34 side chain assumed a “flip-in” orientation that resembled the ApoE3 structure (Figure 2). Therefore, we removed these two structures, leaving two each of the free ApoE3, ApoE4 and holo-ApoE4 structures for further computational analysis, as described below.

### 2.2. Structure-Based Ensemble Virtual Screening Utilizing Complementary Consensus Scoring

**Figure 3:**
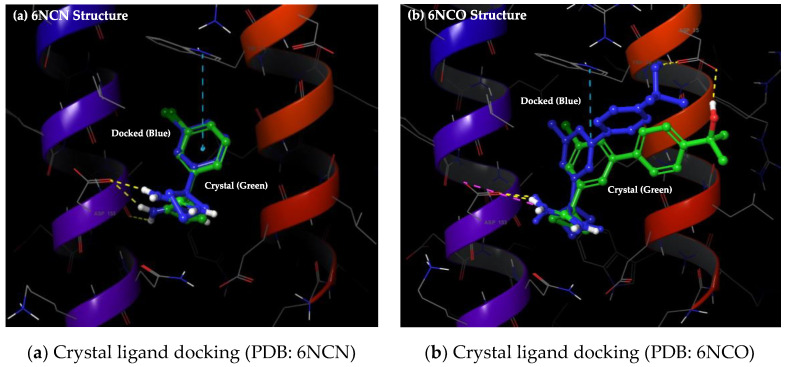
Crystal ligand re-docking: (**a**) PDB ID: 6NCN (ligand PubChem CID: 83673143); (**b**) PDB ID: 6NCO (ligand PubChem CID: 137796780). Crystal ligands (green carbons) and docked (blue).

The ensemble molecular docking studies of bioactive natural compounds from the DCABM-TCM database together with the known ApoE4 stabilizers reported by Petros et al. [25] were carried out against the Tryptophan34 binding pockets of all six ApoE3/E4 structures. The AutoDock Vina [30] docking algorithm was used, whereby the protein remains rigid while the ligand is allowed flexibility. The AutoDock Vina correctly predicted the binding modes of two available crystal ligands (PubChem CIDs: 83673143 and 137796780) with respective ApoE4 structures (PDB IDs: 6NCN and 6NCO). As seen, there was a good overlap between the ligand binding mode observed by X-ray crystallography and the binding mode predicted by AutoDock Vina (Figure 3).

Importantly, the docked ligands replicated important ligand–protein interactions, including pi-pi interaction with Tryptophan34 and H-bond interactions with Aspartate35 and 153 residues. Thus, AutoDock Vina proved well suited for our ensemble docking studies against ApoE proteins and revealed varying binding ranks for the known ApoE4 ligands (Table 1).

Several key observations were made based on these ensemble docking data against ApoE3 and E4 structures. Specifically, all these know ApoE ligands, except the smallest fragment (PubChem CID: 83673143, MW: 208.69 g/mol), showed varying degrees of binding affinity across ApoE3, free-ApoE4, and holo-ApoE4 structures. The ApoE3 and holo-ApoE4 structures, with similar Tryptophan34 sidechain orientation (Figure 1), present similar binding pockets, but only the holo-ApoE4 showed excellent correlation between the AutoDock Vina ranks and the experimental NMR Kd values reported by the AbbVie team [25]. Furthermore, free ApoE4 structures (8CE0 and 8CDY) exhibited negative correlation scores as opposed to the positive correlation scores observed with holo-ApoE4 structures (6NCN and 6NCO). This suggested that molecules that bind well with the holo-ApoE4 pocket bind poorly with the free ApoE4 structure. This is in agreement with the “flip-in” Tryptophan34 orientation in holo-ApoE4 structures (Figure 1) that present a more open, well-defined binding pocket, as opposed to the “flip-out” Tryptophan34 orientation in free-ApoE4 structures closing this pocket. Based on this observation, we devised a new consensus ranking scheme (Equation (1)) involving AutoDock Vina binding ranks against both the free- and holo-ApoE4 structures.

Consensus Rank = [Average holo-ApoE4 Rank (6NCN and 6NCO)]/[Average free-ApoE4 Rank (8CE0 and 8CDY)](1)

Our novel consensus ranking scheme complemented the docking analysis well and further improved the correlation score in comparison with the experimental Kd values to a near-perfect 0.994. Notably, it also significantly improved the overall docking ranks of the more active ApoE4 ligands (Kd values < 30 µM), putting them in the top ~1.6% hitlist. Accordingly, the top 25 novel hits out of ~1250 DCABM-TCM database molecules (top ~2%) obtained through this ensemble docking followed by consensus ranking are listed below (Table 2). Their structures are shown in Supporting Figures S2 and S3.

### 2.3. Ligand-Based Virtual Screening Using 3D Shapes of Most Active ApoE4 Stabilizers

The ligand-centric, shape-based virtual screening has been suggested to obtain more consistent performance across targets in terms of hit rate than other 3D screening methods including structure-based docking and 3D pharmacophore screening [31,32,33]. More importantly, shape similarity techniques often lead to new inhibitors with innovative chemical scaffolds. Therefore, we decided to screen the DCABM-TCM database using the Rapid Overlay of Chemical Structures (ROCS) method [34], which aligns molecules based on shape and/or chemical similarity (ROCS 3.7.0.0. OpenEye, Cadence Molecular Sciences, Santa Fe, NM. http://www.eyesopen.com). In this regard, two crystal structures of small-molecule stabilizers in complex with ApoE4 that present the tentative spatial and chemical requirements necessary for the effective binding of a given molecule to the Tryptophan34 pocket on ApoE4 are currently available (PDB IDs: 6NCN and 6NCO).

**Figure 4:**
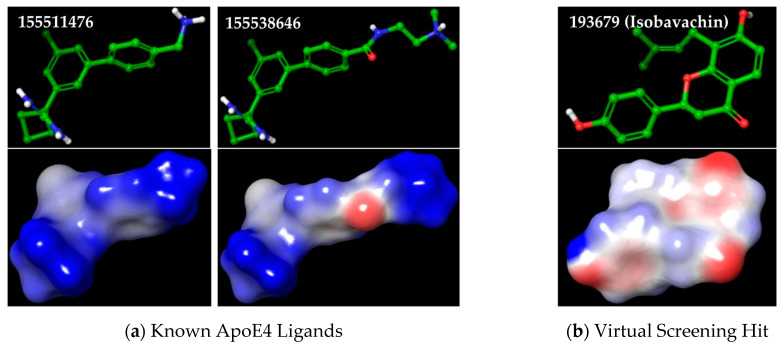
The 3D molecular structures and shapes of known ApoE4 ligands and our top hit molecule: (**a**) two most active ApoE4 ligands (PubChem CIDs: 155511476 and 155538646); (**b**) top virtual screening hit Isobavachin (PubChem CID: 193679).

Unfortunately, the two ligands associated with these two crystal structures (PubChem CIDs: 83673143 and 137796780) are relatively weak ApoE4 ligands possessing poor Kd values to ApoE4 (Table 1). Therefore, we decided to use the two most active ApoE4 ligands (PubChem CIDs: 155511476 and 155538646) with ApoE4 Kd < 5 µM for our ligand-based screening. The best docking conformations of these two ligands, which recapitulated the favorable H-bond and pi–pi interactions in the Tryptophan34 pocket of ApoE4 protein (PDB ID: 6NCO), were used as queries for the 3D shape-based ROCS screening (Figure 4a). The multi-conformer database of TCM-derived molecules was screened to rank the database molecules using ROCS_TanimotoCombo (shape and color) score of the highest-ranking conformer of each compound. These shape-similarity scoring data obtained using two known ApoE4 ligands were then combined to reveal the top 25 molecules with the best ROCS_TanimotoCombo scores (Table 2). As seen, a natural flavonoid, Isobavachin (PubChem CID: 193679), was revealed as the common hit from both the structure- and ligand-based screenings, possessing high docking and 3D shape ranks. The 3D molecular structure and shape of Isobavachin (Figure 4b) mimic those of the two query ApoE4 ligands (Figure 4a), with a ROCS_TanimotoCombo score of 0.904, and possess key H-bond capabilities on two ends. Specifically, the amine groups in the known ApoE4 ligands are mimicked by the hydroxyl groups in Isobavachin. Furthermore, a hydrophobic Cl in known ApoE4 ligands is observed to occupy the Tryptophan34 pocket in ApoE4, which is mimicked by the hydrophobic prenyl group in Isobavachin. Thus, Isobavachin appears to be a top promising hit molecule, presenting a novel scaffold as compared to the known ApoE4 ligands (Tanimoto Similarity 0.03 and 0.04 compared to 155511476 and 155538646, respectively).

### 2.4. Flexible Receptor Docking to Investigate Ligand-Induced Flipping of Tryptophan34 Residue

Molecular modeling studies of a total of 49 hits from docking and shape screening together with 8 known ApoE4 stabilizers from AbbVie [25] were carried out to investigate their binding modes within the Tryptophan34 pocket on free ApoE4 (PDB ID: 8CE0) that has a Tryptophan34 residue in “flip-out” orientation (Figure 1). All these hit compounds are structurally different and thus may not be expected to fit in the ApoE4 pocket in the same way as the known ligands. Therefore, the Induced-Fit Docking (IFD) methodology was used for docking, as it has been shown to be efficient in handling the receptor flexibility for structurally different ligands [35]. The focus was on the ligands capable of replicating the ligand–protein interactions exhibited by the known ApoE4 stabilizers. Specifically, H-bond interactions with Aspratate35 and 153, and most importantly flipping of the Tryptophan34 residue to “flip-in” position, thus opening up a pocket that is to be filled by a suitable hydrophobic group. The IFD studies were successful with all the 8 known ApoE4 ligands successfully changing the orientation of the Tryptophan34 sidechain from the “flip-out” position in free ApoE4 to the “flip-in” position after ligand binding (Table 3). Notably, even the smallest fragment (CID: 83673143) with molecular weight of 208.69 g/mol successfully flipped the Tryptophan34 orientation to resemble that of ApoE3, thus further emphasizing the suitability of the IFD protocol used here. The other desired ligand–protein interactions varied across different ligands, correlating well with their reported experimental binding Kd values. For example, the top 6 out of 8 compounds with Kd values ≤ 30 µM exhibited all the key interactions, while the 2 weakest molecules lacked key H-bond interactions with either Aspartate35 or 153, further highlighting their importance.

**Figure 5:**
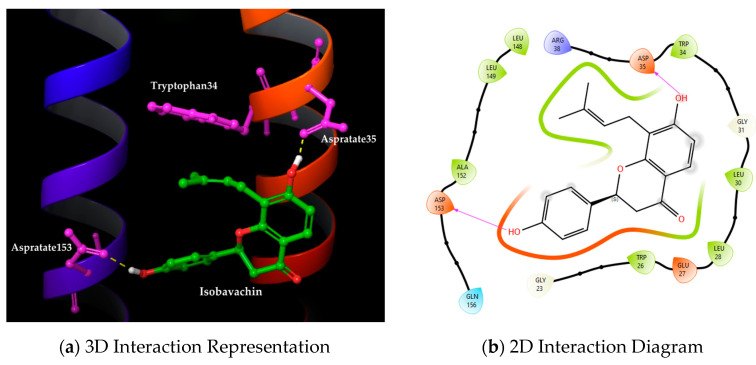
The binding mode of the top-ranking hit molecule Isobavachin (PubChem CID: 193679) obtained through the induced-fit docking: (**a**) Tryptophan34 binding pocket on ApoE4 containing bound ligand; (**b**) 2D ligand–protein interaction diagram.

In this context, out of 49 virtual hits from docking and shape-based screening, only the top common hit Isobavachin exhibited all the key interactions (Figure 5).

It is noteworthy that Isobavachin flipped the Tryptophan34 sidechain to the desired “flip-in” position and formed critical H-bond interactions with Aspartate35 and 153 residues; also, its hydrophobic prenyl perfectly occupied the Tryptophan34 binding pocket on ApoE4. This IFD binding mode of Isobavachin is in accordance with those of the known ApoE4 stabilizers from AbbVie, especially the most active ones with Kd values ranging from ~5–30 µM. Thus, Isobavachin possesses key structural features required to occupy the Tryptophan34 binding pocket on ApoE4, potentially leading to its structure correction and stabilization resembling the ApoE3 protein.

### 2.5. Molecular Dynamics Simulation of Isobavachin-ApoE4 Complex

**Figure 6:**
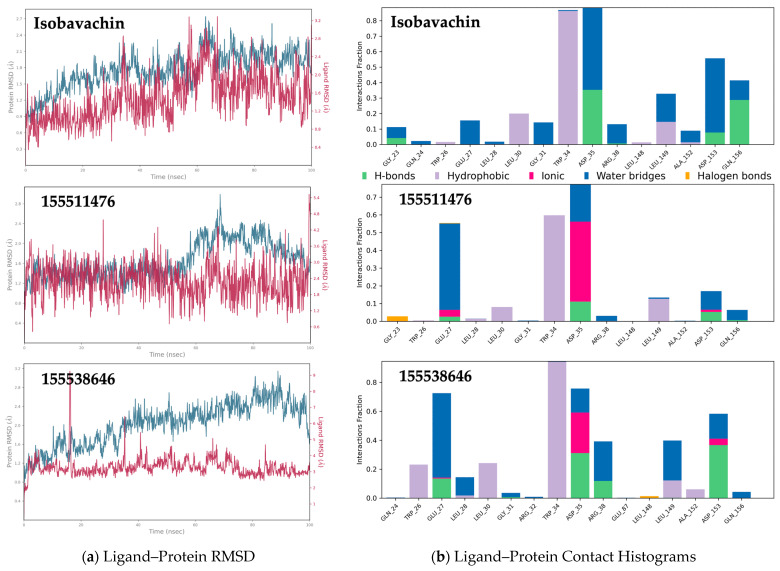
MD simulation data: (**a**) RMSD analysis over 100ns MD simulation of ligand binding with the Tryptophan34 pocket on ApoE4; (**b**) ligand–protein interaction histogram.

The stability of our top virtual hit Isobavachin within the ApoE4 binding pocket was further investigated by carrying out molecular dynamics (MD) simulations. The root-mean-square-distance (RMSD) analysis of Isobavachin over 100 ns MD simulation revealed its stable binding in the Tryptophan34 pocket on ApoE4 (Figure 6a). After an initial configuration change, Isobavachin showed consistent RMSD value around ~2.0 Å throughout the 100 ns simulation period, thus indicating its stable binding with the ApoE4 pocket. Furthermore, the RMSD profile for the Isobavachin-bound ApoE4 backbone was <2.7 Å throughout the simulation, thus further supporting stability of the ligand–protein complex. Indeed, Isobavachin showed relatively much better binding stability with the ApoE4 pocket as compared to the two most active ApoE4 stabilizers reported by AbbVie (CIDs: 155511476 and 155538646). Accordingly, Isobavachin showed a consistently favorable interaction profile with key amino acids such as Tryptophan34 and Aspartate35 and 153 throughout the 100ns simulation interval (Figure 6b).

**Figure 7:**
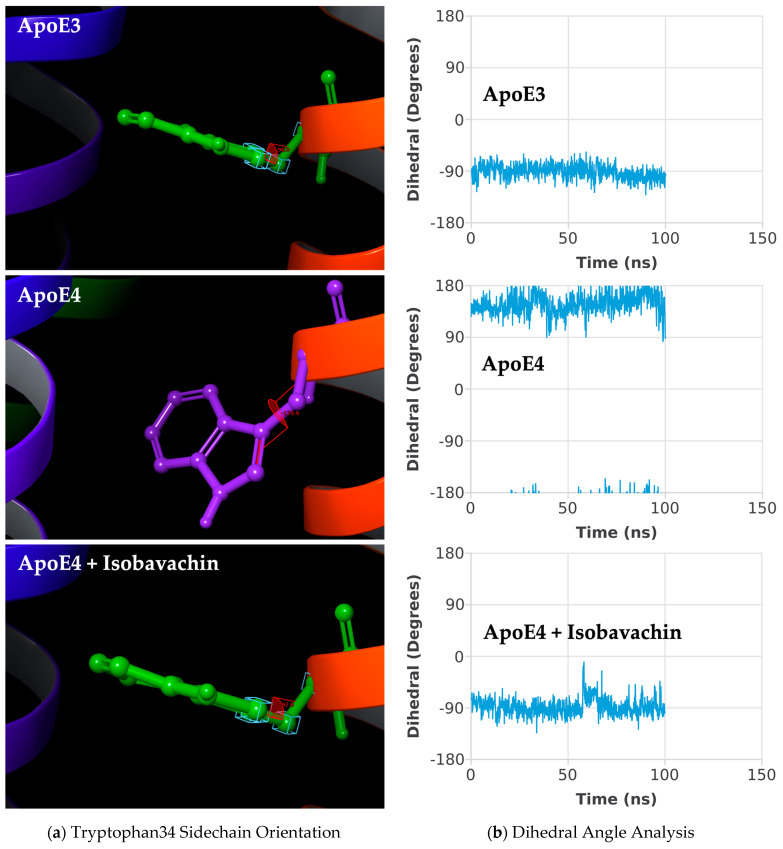
Tryptophan34 sidechain flipping: (**a**) ApoE protein structures averaged across 100 ns MD simulation, showing the representative Tryptophan34 sidechain orientation; (**b**) dihedral angle analysis for the Tryptophan34 residue.

Importantly, Isobavachin effectively changed the orientation of the Tryptophan34 sidechain in ApoE4 to the desired “flip-in” position (Figure 7a), and most importantly, it successfully maintained this orientation throughout the 100 ns MD simulation (Figure 7b). As seen, the orientation of Tryptophan34 sidechain residue averaged over the simulation trajectory remained parallel to the protein axis in free-ApoE4, while it was flipped perpendicular to the protein axis in Isobavachin-bound ApoE4, making it similar to ApoE3. Thus, Isobavachin seems to be an ideal drug candidate for correcting the pathological ApoE4 structure to the beneficial ApoE3 structure.

### 2.6. Biophysical Binding Analysis of Isobavachin and Related Flavonoids with ApoE3 and ApoE4

**Figure 8:**
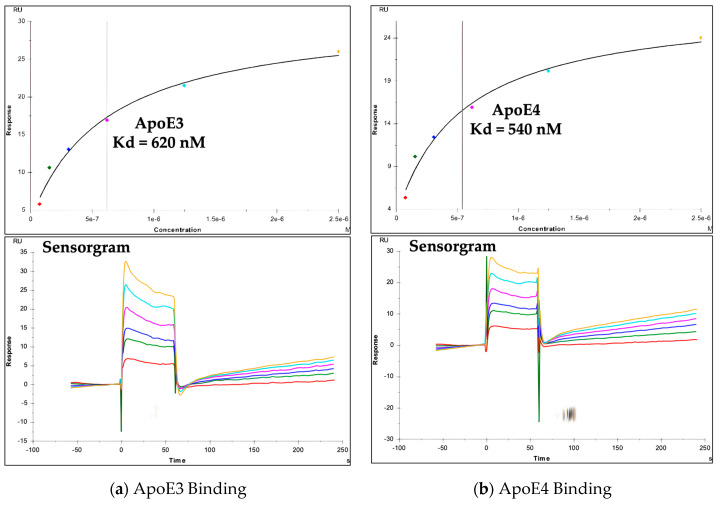
Surface plasmon resonance (SPR) analysis: Isobavachin binding with human recombinant (**a**) ApoE3 and (**b**) ApoE4 proteins.

The top virtual hit Isobavachin was further subjected to surface plasmon resonance (SPR) analysis using Biacore T200 (Cytiva, Marlborough, MA, USA) to determine its binding constants with both the ApoE3 and ApoE4 proteins. Together with the human recombinant ApoE3 and ApoE4 proteins, a negative control protein 2′-deoxynucleoside 5′-phosphate N-hydrolase (His-Sumo-DNPH1) was also included in the SPR analysis. The His-Sumo-DNPH1 is structurally unrelated to ApoE proteins, while having a similar size as ApoE proteins (~30 KDa). In agreement with our extensive computational analysis, Isobavachin showed strong binding with the ApoE4 protein, with a Kd value of ~540 nM (Figure 8).

Isobavachin also showed a similar Kd value with ApoE3 (~620 nM), thus further supporting the potential re-orientation of the Tryptophan34 sidechain residue in ApoE4 resembling that in ApoE3, presenting similar binding pockets for Isobavachin. In contrast, Isobavachin did not bind well with the negative control protein His-Sumo-DNPH1 (Kd value > 100 µM), thus emphasizing its selective binding to ApoE proteins (Table 4).

**Figure 9:**
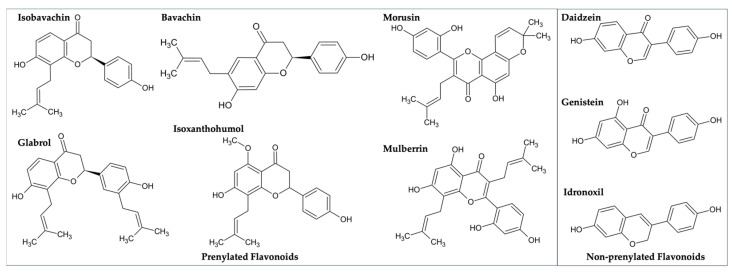
The chemical structures of Isobavachin and other flavonoids tested in the present study.

In addition to Isobavachin, we also tested a few other bioactive purchasable flavonoids for their potential binding with the ApoE3/4 proteins. These included various prenylated (Bavachin, Glabrol, Isoxanthohumol, Morusin, Mulberrin) and non-prenylated (Daidzein, Genistein, Idronoxil) flavonoids (Figure 9).

Only two of these flavonoids, viz. Bavachin and Isoxanthohumol, both prenylated like Isobavachin, showed some binding activity against ApoE3 and ApoE4 proteins (Table 4). Overall, our top virtual hit Isobavachin proved to be the strongest ApoE3/E4 binder.

## 3. Discussion

The present study, through a robust combination of structure- and ligand-based virtual screening methodologies, effectively highlights the potential of natural medicinal compounds from the DCABM-TCM as a rich repository of bioactive compounds for AD drug discovery. The identification of Isobavachin as a natural flavonoid capable of correcting and potentially stabilizing the ApoE4 structure marks a significant step forward in AD research.

ApoE plays a central role in AD pathogenesis, with the “ApoE Cascade Hypothesis” postulating that the isoforms of ApoE (E2, E3, and E4) exert differential effects on AD pathology through complex molecular and cellular mechanisms, contributing to the varying levels of risk and progression of the disease [36]. ApoE4, the strongest genetic risk factor for late-onset AD, promotes Aβ aggregation and deposition, disrupts lipid homeostasis, and exacerbates neuroinflammation and synaptic dysfunction. Mechanistically, ApoE4 has been shown to be less efficient in clearing Aβ compared to ApoE2 and ApoE3, leading to its accumulation in the brain [37]. Furthermore, ApoE4 undergoes proteolytic cleavage, generating toxic fragments that impair mitochondrial function and neuronal viability [38]. This cascade of events accelerates tau pathology and neurodegeneration, creating a feedback loop that perpetuates AD progression [39]. Therapeutically, targeting the ApoE cascade through stabilizing the ApoE4 structure (both domain interaction and Trptophan34 sidechain orientation), modulating its lipidation, or mitigating its pathological interactions offers promising avenues for curative interventions in AD [19,25,40,41].

By stabilizing the ApoE4 structure through Tryptophan34 re-orientation, Isobavachin may mitigate its toxic gain-of-function properties and effectively reduce its contribution to neurodegenerative processes in AD. Importantly, Isobavachin is a bioactive compound, a naturally occurring prenylated flavonoid derived primarily from the seeds of Psoralea corylifolia (commonly known as Babchi). Prenylated flavonoids have been shown to exhibit wide-ranging medicinal properties, such as anti-oxidant, anti-inflammatory, anti-cancer, anti-diabetic, anti-microbial, and neuroprotective effects, making it an attractive candidate for therapeutic applications [42]. It is noteworthy that Isobavachin has been shown to readily penetrate the blood–brain barrier (BBB), thereby enabling its potential to directly affect central nervous system (CNS) pathologies, including AD [43]. Overall, the natural origin of Isobavachin and its bioavailability as a TCM-derived compound, coupled with its ApoE4-centric effects outlined in this study, underscores its translational potential as a favorable therapeutic agent against AD.

Our combined molecular docking and 3D shape screening protocol successfully identified Isobavachin as the top hit, which was then experimentally confirmed to be a potent ApoE ligand. Importantly, the present study underscores the importance of selecting appropriate protein structures for virtual screening of potential ApoE4 stabilizers that act through ligand-induced conformation changes. Our decision to exclude atypical ApoE structures based on Tryptophan34 sidechain orientation resulted in a refined dataset of ApoE3 and ApoE4 structures. This approach in turn ensured the enhanced reliability of computational predictions, as supported by strong correlations between experimental and predicted binding affinities for known ApoE ligands (Table 1). Moreover, our novel consensus ranking scheme (Equation (1)) further improved predictive accuracy by emphasizing the distinct binding properties of free- and holo-ApoE4 structures, yielding a near-perfect correlation score of 0.994 with the experimental Kd values. These encouraging results demonstrate that the adopted integrated virtual and experimental protocol, together with our innovative consensus ranking scheme, may prove to be a useful tool for the identification of novel hits that can be developed into potent ApoE-targeted therapies for AD.

In this context, further experimental validation of Isobavachin’s efficacy in cellular and in vivo models of AD is warranted to further confirm its therapeutic potential against various AD-related changes, including Aβ deposition, NFT formation, and region-specific neurodegeneration [44,45]. In addition, medicinal chemistry structural optimizations to enhance binding affinity and specificity for the Tryptophan34 pocket could also be explored to develop novel analogs of Isobavachin. For example, a possible replacement of the prenyl group in Isobavachin with other hydrophobic groups may be explored for compounds with improved drug-like properties. Moreover, the replacement of hydroxyl groups with other H-bonding moieties such as amine groups may lead to compounds with improved bioavailability, metabolic stability, receptor binding affinity, and overall therapeutic efficacy [46]. Accordingly, future in vivo studies exploring the pharmacokinetics, pharmacodynamics, and safety profiles of Isobavachin and its potent analogs will be crucial for their advancement into preclinical and clinical development.

In summary, the findings of this study establishing Isobavachin as a potential ApoE4 structure corrector may offer significant implications for the development of disease-modifying therapies for AD.

## 4. Materials and Methods

### 4.1. Ensemble Molecular Docking

The DCABM-TCM database was prepared for docking by extracting SMILES from http://bionet.ncpsb.org.cn/dcabm-tcm/ (accessed on 25 June 2024) [26]. The database included ~1740 bioavailable TCM compounds detected in the blood, out of which ~500 molecules with high molecular weights were removed. The 3D conformers were generated for the remaining molecules using OMEGA 6.0.0.0 3D conformer generator [47] from OpenEye, Cadence Molecular Sciences, Santa Fe, NM, http://www.eyesopen.com. The ensemble docking of this drug database was carried out using two each crystal structures of the ApoE3 (PDB: 1NFN, 1BZ4), free ApoE4 (PDB: 8CE0, 8CDY), and holo-ApoE4 (PDB: 6NCN, 6NCO) proteins downloaded from the protein data bank (PDB) [48]. These ApoE protein structures were processed using the AutoDock Tools utility [49], whereby bound ligands and water molecules were removed, polar hydrogens were added, non-polar hydrogens were merged, and Gasteiger charges were assigned for all the atoms in the proteins. The ApoE proteins and ligand atoms were converted into PDBQT format. The AutoDock Vina docking algorithm [30] was then used to carry out structure-based docking of the DCABM-TCM molecules to the Tryptophan34 binding site on ApoE proteins. All ApoE structures were overlapped on to the 6NCO crystal structure, and thus the same search space coordinates were used for docking into all these ApoE structures [Center—X: 2.30, Y: −12.59, Z: 11.90; Size—X: 25 Å, Y: 25 Å, Z: 25 Å]. Default docking parameters were used, and the docked ligands were ranked according to their best docking score values. A novel consensus scoring utilizing docking scores against both the free- and holo-ApoE4 structures was used to shortlist the top 25 hits for further analysis.

### 4.2. Ligand-Based Virtual Screening

Ligand-based virtual screening was performed using ROCS 3.4.1.0 3D shape-based method [34] from OpenEye, Cadence Molecular Sciences, Santa Fe, NM, http://www.eyesopen.com. The DCABM-TCM database was processed using OMEGA 6.0.0.0 using default parameters, which generated ~65K 3D conformers. ROCS was used to explore this 3D multi-conformer database for molecules with similar shape and color as the best docked conformations of the two most active ApoE4 stabilizers reported by AbbVie Pharmaceuticals (PubChem CIDs: 155511476 and 155538646). The database compounds were ranked by the TanimotoCombo scores for their highest-ranking conformers. The 3D shape-similarity scoring data thus obtained across these two known ApoE4 ligands were then combined to short-list the top 25 molecules with the best TanimotoCombo scores.

### 4.3. Induced-Fit Docking (IFD)

The flexible-receptor docking of top structure- and ligand-based virtual screening hits was carried out within the Tryptophan34 residue site of the free ApoE4 protein crystal structure (PDB ID: 8CE0). The IFD protocol was run with default parameters from the Schrodinger’s Maestro graphical user interface. The first stage of IFD protocol involved extended sampling using Glide docking algorithm to generate 80 initial poses through a softened-potential docking step, which allows for the tolerance of more steric clashes as compared to a normal, rigid-receptor docking protocol. The second stage then involved protein refinement using the Prime module to allow for the active site conformational change around 10 Å of the initial ligand poses. Herein, energy minimization was carried out using OPLS4 force field and Prime’s implicit solvent model. Finally, the active site was further refined by Prime followed by redocking by Glide XP scoring function, leading to the generation of the final IFD models. The top 10 IFD poses for each molecule were graphically visualized using Maestro and the best conformation for each molecule based on the observed binding mode and the IFD score was chosen for further analysis including molecular dynamics (MD) simulations as described below.

### 4.4. Molecular Dynamics Studies

The binding stability of our top hits was investigated using the molecular dynamics (MD) simulations of the respective ligand–protein complexes using Schrodinger’s Desmond MD module. Briefly, the top binding conformations from the induced-fit docking were chosen as the starting conformations for the MD simulations. The ligand interactions were modeled with the OPLS4 force field. The ligand–protein docking complexes were solvated using TIP5P water model in an orthorhombic boundary box of 10 Å size in all three directions, followed by their neutralization with appropriate number of Cl- ions. Also, salt was added at a concentration of 0.15 M to simulate the physiological salt concentration. Default parameters in Desmond including OPLS4 force field were utilized to equilibrate this molecular system. Finally, each of the equilibrated systems was subjected to 100 ns of MD simulations at constant temperature and pressure of 300 K and 1 atm, respectively. The atom coordinates were recorded at every 100 ps for the follow-up simulation analyses. The root mean square deviation (RMSD) for the proteins and the docked ligands were calculated over the entire simulation trajectory with reference to their respective first frames. The proteins’ interactions with the ligand were normalized over the course of the 100 ns trajectory, with the score value depicted in the stacked bar chart indicating the percentage of the simulation time a specific interaction is maintained. The representative structures averaged over the entire simulation time were obtained and the variance of dihedral angles for the Tryptophan34 sidechain residue was plotted across the simulation trajectory.

### 4.5. Surface Plasmon Resonance (SPR)

The biophysical binding analysis of ligands with ApoE3 and ApoE4 proteins was carried out using the SPR technique. Human recombinant ApoE3 or ApoE4 proteins were purchased from PeproTech (Cranbury, NJ, USA) and reconstituted according to the manufacturer’s instructions. The negative control protein was His-Sumo-DNPH1 (2′-deoxynucleoside 5′-phosphate N-hydrolase 1), produced in-house in *E. coli* by The Wistar Institute, Philadelphia. The proteins were covalently attached to a carboxymethyl dextran sensor chip (CMD700M, from Xantec bioanalytics, Düsseldorf, Germany) using standard amine coupling in HEPES buffered saline. The chip is first conditioned for 180 s at 10 µL/min with 1 M NaCl, 0.1 mM Na Borate, pH 9.0, and then activated with 250 mM EDC/100 mM NHS for 600 s at 10 µL/min. The ApoE3/4 and DNPH1 proteins diluted in 10 mM sodium acetate (pH 5.5 and 4.0, respectively) were injected and immobilized on respective flow cells. After immobilization, the remaining activated sites were blocked with 1 M ethanolamine at 10 µL/min for 180 s. Test compounds (purchased from Cayman Chemical (Ann Arbor, MI, USA) and MedChemExpress (Monmouth Junction, NJ, USA)) were first diluted in pure DMSO, which were then further diluted in the running buffer without DMSO (10 mM HEPES, pH 7.4, 150 mM NaCl, 0.005% Tween20) to match the DMSO concentrations of the sample and the running buffer. The association and dissociation times were 60s and 120s, respectively, and the flow rate was 30 µL/min. After each compound injection, the needle was washed in 50% DMSO to reduce compound carry-over. Solvent correction cycles were also included to correct for bulk refractive index changes associated with DMSO.

## 5. Conclusions

The present study demonstrates the discovery of Isobavachin, a natural flavonoid, as a promising ApoE4 structure corrector for Alzheimer’s disease (AD). Through an integrated computational and experimental approach, we identified Isobavachin as a top hit that effectively binds to the Tryptophan34 pocket of ApoE4 and induces the desired conformational shift towards an ApoE3-like structure. The observed ligand-induced flipping of the Tryptophan34 sidechain, along with key protein–ligand interactions, highlights the potential of Isobavachin in addressing the aberrant structure and neurotoxic downstream effects of ApoE4.

This work underscores the utility of natural products, particularly flavonoids, as valuable scaffolds for AD drug discovery. By employing complementary structure-based docking, ligand-based virtual screening, and molecular dynamics simulations, followed by biophysical validation, we have established a comprehensive framework for identifying and characterizing ApoE4 stabilizers. Importantly, the study validates the viability of the combined screening protocol to explore other bioactive compound libraries for novel ApoE4-correcting small molecules.

The results lay a strong foundation for further preclinical investigations into Isobavachin and similar flavonoids as potential disease-modifying agents. Future studies will focus on comprehensive structure–activity relationship (SAR) studies optimizing the chemical scaffold of Isobavachin to enhance its potency, selectivity, and pharmacokinetic properties. Additionally, in vivo validation in AD models is necessary to evaluate its therapeutic efficacy and safety profile. By targeting ApoE4’s structural instability, this research opens new avenues for the development of effective and targeted treatments for AD, addressing a critical unmet need in the field.

## Figures and Tables

**Table 1:** The AutoDock Vina binding ranks for known ApoE4 ligands against ApoE3, free ApoE4, and holo-ApoE4.

PubChem CID	NMR Kd (µM)	ApoE3 (1NFN)	ApoE3 (1BZ4)	ApoE4 (8CE0)	ApoE4 (8CDY)	Holo-ApoE4 (6NCN)	Holo-ApoE4 (6NCO)	Consensus Rank
83673143	900 ± 108	1027	1013	1029	993	733	853	286
155530661	230 ± 24	328	490	185	204	519	572	1167
137796780	30 ± 4	527	565	242	298	65	45	28
155563897	20 ± 3	426	208	186	628	60	18	9
155552638	20 ± 4	444	400	660	664	109	76	14
155557185	8 ± 3	471	652	519	505	61	25	6
155511476	<5	470	651	827	774	111	95	11
155538646	<5	594	611	659	744	116	148	21
Correlation Score ^1^		0.839	0.723	−0.546	−0.738	0.953	0.973	**0.996**

^1^ Correlation scores between AutoDock Vina ranks and experimental Kd values for ApoE4 binding. Shown bold is the highest correlation score based on our consensus scoring scheme.

**Table 2:** Top 25 hits each from the structure-based ensemble docking and shape-based screening.

Sr. No.	Consensus Docking Hits (PubChem CIDs)	Consensus ROCS Hits (PubChem CIDs)
1	100781	53266
2	161294	160487
3	91510	5320382
4	**193679 ^1^**	114829
5	441805	5282073
6	11556558	632135
7	94320	**193679 ^1^**
8	9974201	46886723
9	10114	115067
10	71773126	5281601
11	163184367	5316844
12	14077830	92775
13	9983614	5280443
14	11003773	96539
15	64945	5281617
16	102501232	5322078
17	73347309	10450045
18	9548703	198910
19	14463159	5319081
20	91471	5320053
21	5380976	11601633
22	114850	439246
23	163005195	5318980
24	92097	5281698
25	151529	72303

^1^ The combined docking- and shape-based screening revealed 193679 as a common hit (shown bold).

**Table 3:** The ligand–protein interaction characteristics obtained for the IFD binding modes of known ApoE4 ligands. The ligand–protein interaction diagrams are presented in the Supporting Figure S4.

PubChem CID	NMR Kd (µM)	Tryptophan34 Orientation	Aspartate35 Interaction	Aspartate153 Interaction	Hydrophobic Pocket Filling
83673143	900 ± 108	Flip-in	––––	H-bond	Cl atom
155530661	230 ± 24	Flip-in	H-bond	––––	Cl atom
137796780	30 ± 4	Flip-in	H-bond	H-bond	Cl atom
155563897	20 ± 3	Flip-in	H-bond	H-bond	Cl atom
155552638	20 ± 4	Flip-in	H-bond	H-bond	Cl atom
155557185	8 ± 3	Flip-in	H-bond	H-bond	Cl atom
155511476	<5	Flip-in	H-bond	H-bond	Cl atom
155538646	<5	Flip-in	H-bond	H-bond	Cl atom

**Table 4:** SPR binding data for Isobavachin and other bioactive flavonoids.

Name	PubChem CID	ApoE3 Kd (µM)	ApoE4 Kd (µM)	DNPH1 Kd (µM)
Isobavachin	193679	0.62	0.54	>100
Bavachin	14236566	59	64	No binding
Daidzein	5281708	>100	>100	>100
Genistein	5280961	>100	>100	>100
Glabrol	11596309	>100	77	>100
Idronoxil	219100	>100	>100	>100
Isoxanthohumol	513197	38	40	>100
Morusin	5281671	>100	>100	69
Mulberrin	5481958	>100	>100	>100

## Data Availability

The data are available on request from the corresponding author.

## Supplementary Materials

The following supporting information can be downloaded at https://www.mdpi.com/article/10.3390/molecules30040940/s1. Figure S1: The 2D chemical structures of ApoE4 ligands reported by the AbbVie Pharmaceuticals; Figure S2: The 2D chemical structures of top 25 docking hits; Figure S3: The 2D chemical structures of top 25 ROCS hits; Figure S4: The induced-fit docking modes of 8 known ApoE4 ligands reported by the AbbVie Pharmaceuticals; Table S1: Top 25 molecules selected through structure-based docking; Table S2: Top 25 molecules selected through ligand-based 3D shape screening.

## References

[1] (2024). 2024 Alzheimer’s disease facts and figures. Alzheimers Dement..

[2] Soria Lopez J.A., Gonzalez H.M., Leger G.C. (2019). Chapter 13-Alzheimer’s disease. Handb. Clin. Neurol..

[3] Young-Pearse T.L., Lee H., Hsieh Y.C., Chou V., Selkoe D.J. (2023). Moving beyond amyloid and tau to capture the biological heterogeneity of Alzheimer’s disease. Trends Neurosci..

[4] Patil S., Chan C. (2005). Palmitic and stearic fatty acids induce Alzheimer-like hyperphosphorylation of tau in primary rat cortical neurons. Neurosci. Lett..

[5] Patil S., Sheng L., Masserang A., Chan C. (2006). Palmitic acid-treated astrocytes induce BACE1 upregulation and accumulation of C-terminal fragment of APP in primary cortical neurons. Neurosci. Lett..

[6] Patil S., Melrose J., Chan C. (2007). Involvement of astroglial ceramide in palmitic acid-induced Alzheimer-like changes in primary neurons. Eur. J. Neurosci..

[7] Haass C., Selkoe D.J. (2007). Soluble protein oligomers in neurodegeneration: Lessons from the Alzheimer’s amyloid beta-peptide. Nat. Rev. Mol. Cell Biol..

[8] Walsh D.M., Selkoe D.J. (2020). Amyloid β-protein and beyond: The path forward in Alzheimer’s disease. Curr. Opin. Neurobiol..

[9] Hong S., Beja-Glasser V.F., Nfonoyim B.M., Frouin A., Li S., Ramakrishnan S., Merry K.M., Shi Q., Rosenthal A., Barres B.A. (2016). Complement and microglia mediate early synapse loss in Alzheimer mouse models. Science.

[10] Karran E., Mercken M., Strooper B. (2011). The amyloid cascade hypothesis for Alzheimer’s disease: An appraisal for the development of therapeutics. Nat. Rev. Drug Discov..

[11] Mullard A. (2023). FDA approves second anti-amyloid antibody for Alzheimer disease. Nat. Rev. Drug Discov..

[12] Cummings J. (2023). Anti-Amyloid Monoclonal Antibodies are Transformative Treatments that Redefine Alzheimer’s Disease Therapeutics. Drugs.

[13] Knopman D.S., Amieva H., Petersen R.C., Chételat G., Holtzman D.M., Hyman B.T., Nixon R.A., Jones D.T. (2021). Alzheimer disease. Nat. Rev. Dis. Primers.

[14] Corder E.H., Saunders A.M., Strittmatter W.J., Schmechel D.E., Gaskell P.C., Small G.W., Roses A.D., Haines J.L., Pericak-Vance M.A. (1993). Gene dose of apolipoprotein E type 4 allele and the risk of Alzheimer’s disease in late onset families. Science.

[15] Mahley R.W., Huang Y. (1999). Apolipoprotein E: From atherosclerosis to Alzheimer’s disease and beyond. Curr. Opin. Lipidol..

[16] Patil S.P., DiFlumeri J., Wellington J., Fattakhova E., Oravic M. (2022). Alzheimer’s neuroinflammation: A crosstalk between immune checkpoint PD1-PDL1 and ApoE-Heparin interactions?. Med. Hypotheses.

[17] Liu C.C., Kanekiyo T., Xu H., Bu G. (2013). Apolipoprotein E and Alzheimer disease: Risk, mechanisms and therapy. Nat. Rev. Neurol..

[18] Patil S.P., Ballard R., Sanchez S., Osborn J., Santangelo D. (2012). ApoE: The link between Alzheimer’s-related glucose hypometabolism and Aβ deposition?. Med. Hypotheses.

[19] Patil S.P., Kuehn B.R. (2024). Discovery of Small Molecule Glycolytic Stimulants for Enhanced ApoE Lipidation in Alzheimer’s Disease Cell Model. Pharmaceuticals.

[20] Fernández-Calle R., Konings S.C., Frontiñán-Rubio J., García-Revilla J., Camprubí-Ferrer L., Svensson M., Martinson I., Boza-Serrano A., Venero J.L., Nielsen H.M. (2022). APOE in the bullseye of neurodegenerative diseases: Impact of the APOE genotype in Alzheimer’s disease pathology and brain diseases. Mol. Neurodegener..

[21] Yamazaki Y., Zhao N., Caulfield T.R., Liu C.C., Bu G. (2019). Apolipoprotein E and Alzheimer disease: Pathobiology and targeting strategies. Nat. Rev. Neurol..

[22] Mahley R.W. (2016). Central Nervous System Lipoproteins: ApoE and Regulation of Cholesterol Metabolism. Arter. Thromb. Vasc. Biol..

[23] Wang C., Najm R., Xu Q., Jeong D.E., Walker D., Balestra M.E., Yoon S.Y., Yuan H., Li G., Miller Z.A. (2018). Gain of toxic apolipoprotein E4 effects in human iPSC-derived neurons is ameliorated by a small-molecule structure corrector. Nat. Med..

[24] Mahley R.W., Huang Y. (2012). Small-molecule structure correctors target abnormal protein structure and function: Structure corrector rescue of apolipoprotein E4-associated neuropathology. J. Med. Chem..

[25] Petros A.M., Korepanova A., Jakob C.G., Qiu W., Panchal S.C., Wang J., Dietrich J.D., Brewer J.T., Pohlki F., Kling A. (2019). Fragment-Based Discovery of an Apolipoprotein E4 (apoE4) Stabilizer. J. Med. Chem..

[26] Liu X., Liu J., Fu B., Chen R., Jiang J., Chen H., Li R., Xing L., Yuan L., Chen X. (2023). DCABM-TCM: A Database of Constituents Absorbed into the Blood and Metabolites of Traditional Chinese Medicine. J. Chem. Inf. Model..

[27] Newman D.J., Cragg G.M. (2016). Natural Products as Sources of New Drugs from 1981 to 2014. J. Nat. Prod..

[28] Patil S.P., Maki S., Khedkar S.A., Rigby A.C., Chan C. (2010). Withanolide A and asiatic acid modulate multiple targets associated with amyloid-beta precursor protein processing and amyloid-beta protein clearance. J. Nat. Prod..

[29] Patil S.P., Tran N., Geekiyanage H., Liu L., Chan C. (2013). Curcumin-induced upregulation of the anti-tau cochaperone BAG2 in primary rat cortical neurons. Neurosci. Lett..

[30] Trott O., Olson A.J. (2010). AutoDock Vina: Improving the speed and accuracy of docking with a new scoring function, efficient optimization, and multithreading. J. Comput. Chem..

[31] Lee H.S., Choi J., Kufareva I., Abagyan R., Filikov A., Yang Y., Yoon S. (2008). Optimization of high throughput virtual screening by combining shape-matching and docking methods. J. Chem. Inf. Model..

[32] Patil S.P., Ballester P.J., Kerezsi C.R. (2014). Prospective virtual screening for novel p53-MDM2 inhibitors using ultrafast shape recognition. J. Comput. Aided Mol. Des..

[33] Fattakhova E., Hofer J., DiFlumeri J., Cobb M., Dando T., Romisher Z., Wellington J., Oravic M., Radnoff M., Patil S.P. (2021). Identification of the FDA-Approved Drug Pyrvinium as a Small-Molecule Inhibitor of the PD-1/PD-L1 Interaction. ChemMedChem.

[34] Hawkins P.C., Skillman A.G., Nicholls A. (2007). Comparison of shape-matching and docking as virtual screening tools. J. Med. Chem..

[35] Sherman W., Day T., Jacobson M.P., Friesner R.A., Farid R. (2006). Novel procedure for modeling ligand/receptor induced fit effects. J. Med. Chem..

[36] Martens Y.A., Zhao N., Liu C.C., Kanekiyo T., Yang A.J., Goate A.M., Holtzman D.M., Bu G. (2022). ApoE Cascade Hypothesis in the pathogenesis of Alzheimer’s disease and related dementias. Neuron.

[37] Huang Y., Mahley R.W. (2014). Apolipoprotein E: Structure and function in lipid metabolism, neurobiology, and Alzheimer’s diseases. Neurobiol. Dis..

[38] Morrow J.A., Hatters D.M., Lu B., Hochtl P., Oberg K.A., Rupp B., Weisgraber K.H. (2002). Apolipoprotein E4 forms a molten globule. A potential basis for its association with disease. J. Biol. Chem..

[39] Holtzman D.M., Herz J., Bu G. (2012). Apolipoprotein E and apolipoprotein E receptors: Normal biology and roles in Alzheimer disease. Cold Spring Harb. Perspect. Med..

[40] Nemergut M., Marques S.M., Uhrik L., Vanova T., Nezvedova M., Gadara D.C., Jha D., Tulis J., Novakova V., Planas-Iglesias J. (2023). Domino-like effect of C112R mutation on ApoE4 aggregation and its reduction by Alzheimer’s Disease drug candidate. Mol. Neurodegener..

[41] Chen H.K., Liu Z., Meyer-Franke A., Brodbeck J., Miranda R.D., McGuire J.G., Pleiss M.A., Ji Z.S., Balestra M.E., Walker D.W. (2012). Small molecule structure correctors abolish detrimental effects of apolipoprotein E4 in cultured neurons. J. Biol. Chem..

[42] Morante-Carriel J., Nájera H., Samper-Herrero A., Živković S., Martínez-Esteso M.J., Martínez-Márquez A., Sellés-Marchart S., Obrebska A., Bru-Martínez R. (2024). Therapeutic Potential of Prenylated Flavonoids of the Fabaceae Family in Medicinal Chemistry: An Updated Review. Int. J. Mol. Sci..

[43] Yang Y.F., Zhang Y.B., Chen Z.J., Zhang Y.T., Yang X.W. (2018). Plasma pharmacokinetics and cerebral nuclei distribution of major constituents of Psoraleae fructus in rats after oral administration. Phytomedicine.

[44] Sullivan P.M., Mace B.E., Maeda N., Schmechel D.E. (2004). Marked regional differences of brain human apolipoprotein E expression in targeted replacement mice. Neuroscience.

[45] Patil S., Balu D., Melrose J., Chan C. (2008). Brain region-specificity of palmitic acid-induced abnormalities associated with Alzheimer’s disease. BMC Res. Notes.

[46] Di L., Kerns E., Carter E. (2009). Drug-Like Property Concepts in Pharmaceutical Design. Curr. Pharm. Des..

[47] Hawkins P.C.D., Skillman A.G., Warren G.L., Ellingson B.A., Stahl M.T. (2010). Conformer Generation with OMEGA: Algorithm and Validation Using High Quality Structures from the Protein Databank and the Cambridge Structural Database. J. Chem. Inf. Model..

[48] Berman H.M., Battistuz T., Bhat T.N., Bluhm W.F., Bourne P.E., Burkhardt K., Feng Z., Gilliland G.L., Iype L., Jain S. (2002). The Protein Data Bank. Acta Crystallogr. D Biol. Crystallogr..

[49] Morris G.M., Huey R., Lindstrom W., Sanner M.F., Belew R.K., Goodsell D.S., Olson A.J. (2009). AutoDock4 and AutoDockTools4: Automated docking with selective receptor flexibility. J. Comput. Chem..

