# Supplementary material for: Discovery of Isobavachin, a Natural Flavonoid, as an Apolipoprotein E4 (ApoE4) Structure Corrector for Alzheimer’s Disease
Source: Molecules. 2025 Feb 18;30(4):940. doi: 10.3390/molecules30040940 (PMC11858207; doi:10.3390/molecules30040940)
Supplement: Supplementary file 1 [file molecules-30-00940-s001.zip › molecules-3428912-supplementary.pdf]

Discovery of Isobavachin, a Natural Flavonoid, as an Apolipoprotein E4 (ApoE4) Structure Corrector for Alzheimer's Disease

Sachin P. Patil [1,2,*], Bella R. Kuehn [1], Christina McCullough [1], Dean Bates [1], Hadil Hazim [1], Mamadou Diallo [1] and Naomie Francois[1]

[1] NanoBio Laboratory, Widener University, Chester, PA 19013, USA
[2] Department of Chemical Engineering, Widener University, Chester, PA 19013, USA
* Correspondence: spatil@widener.edu

Supporting Information

Supporting Figure S1. The 2D chemical structures of ApoE4 ligands reported by the AbbVie Pharmaceuticals [1].

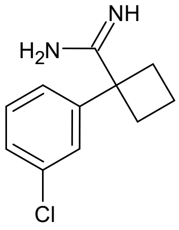

CID_83673143

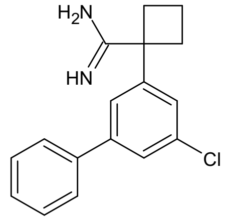

CID_155530661

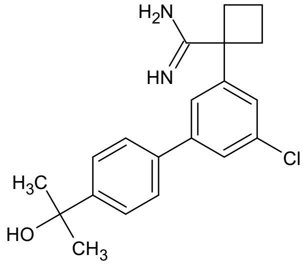

CID_137796780

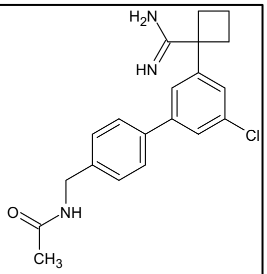

CID_137796780

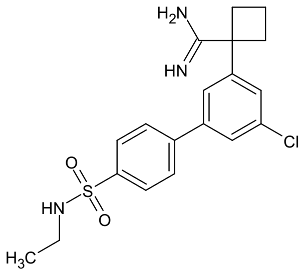

CID_155552638

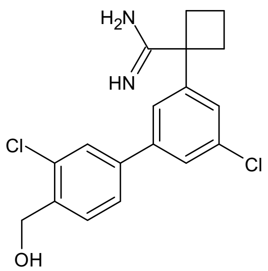

CID_155557185

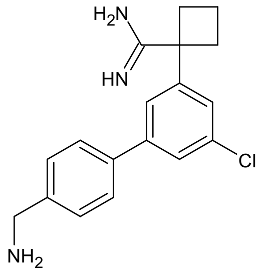

CID_155511476

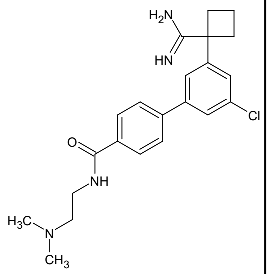

CID_155538646

Supporting Figure S2. The 2D chemical structures of top 25 docking hits.

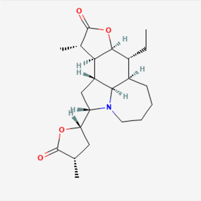
CID_100781

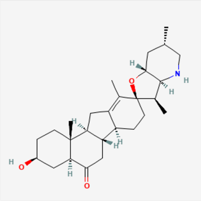
CID_161294

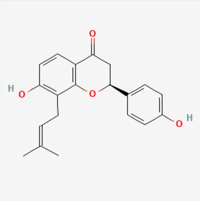
CID_193679

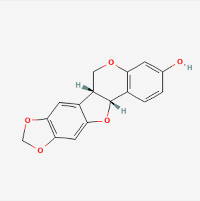
CID_91510

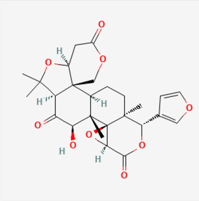
CID_441805

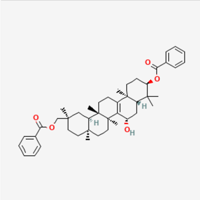
CID_11556558

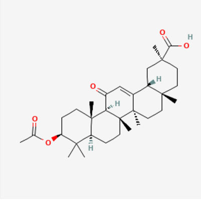
CID_94320

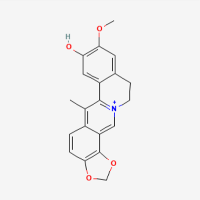
CID_9974201

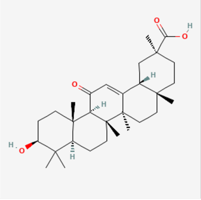
CID_10114

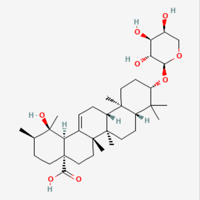
CID_71773126

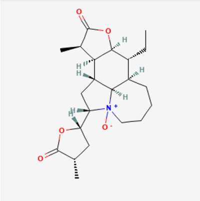
CID_163184367

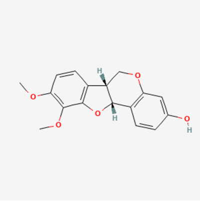
CID_14077830

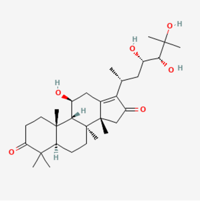
CID_9983614

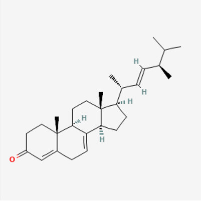
CID_11003773

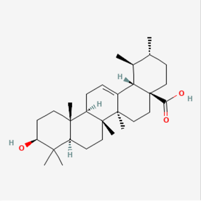
CID_64945

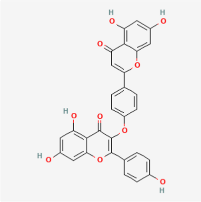
CID_102501232

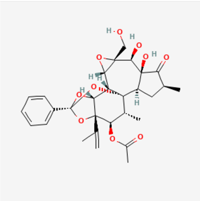
CID_73347309

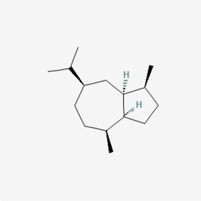
CID_9548703

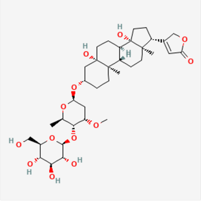
CID_14463159

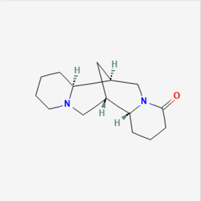
CID_91471

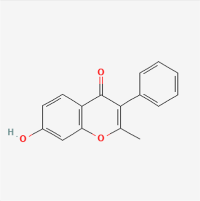
CID_5380976

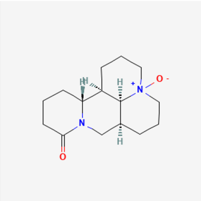
CID_114850

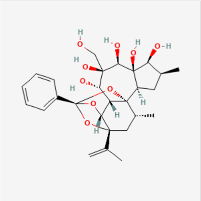
CID_163005195

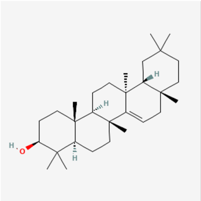
CID_92097

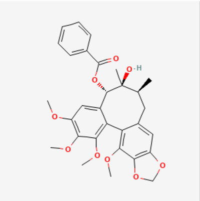
CID_151529

Supporting Figure S3. The 2D chemical structures of top 25 ROCS hits.

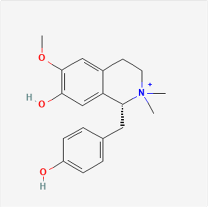

CID_53266

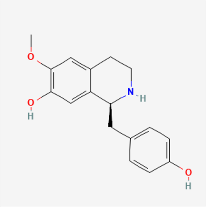

CID_160487

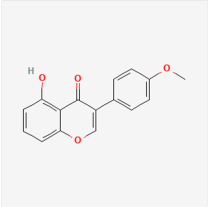

CID_5320382

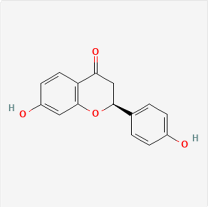

CID_114829

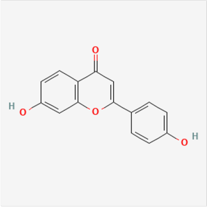

CID_5282073

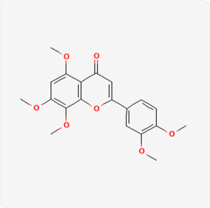

CID_632135

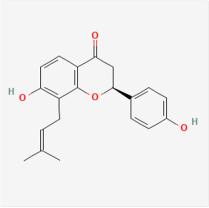

CID_193679

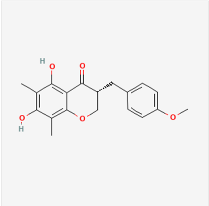

CID_46886723

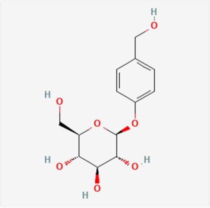

CID_115067

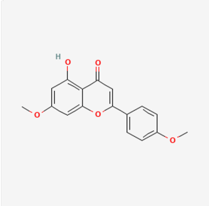

CID_5281601

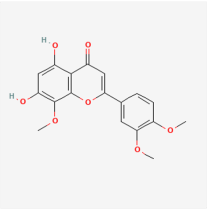

CID_5316844

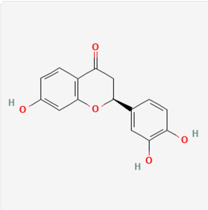

CID_92775

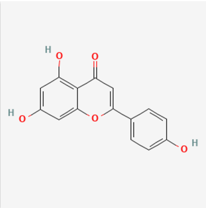

CID_5280443

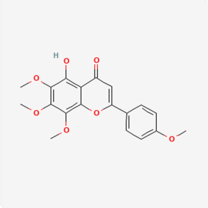

CID_96539

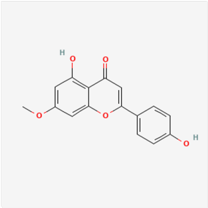

CID_5281617

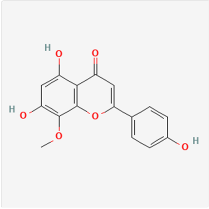

CID_5322078

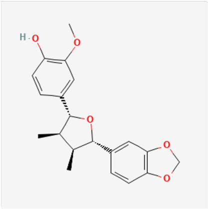

CID_10450045

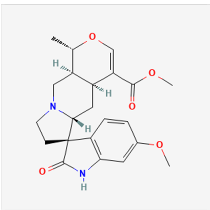

CID_198910

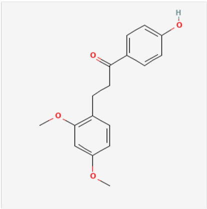

CID_5319081

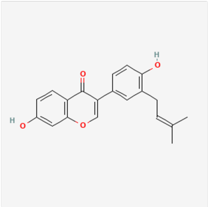

CID_5320053

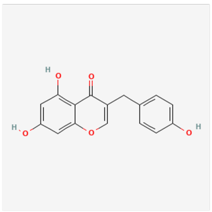

CID_11601633

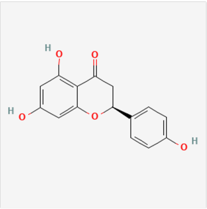

CID_439246

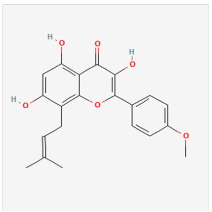

CID_5318980

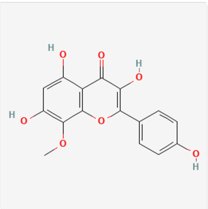

CID_5281698

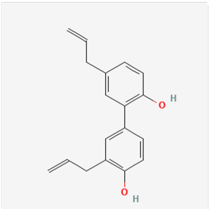

CID_72303

Supporting Table S1. Top 25 molecules selected through structure-based docking and ranked based on their consensus ranks utilizing relative docking scores against holo-ApoE4 structures (PDB IDs: 6NCN and 6NCO) and free ApoE4 structures (PDB IDs: 8CE0, 8CDY).

Ligand ID	Vina Rank (6NCO)	Vina Rank (6NCN)	Vina Rank (8CE0)	Vina Rank (8CDY)	Consensus Rank
100781	4	5	51	279	0.027272727
161294	1	2	38	43	0.037037037
91510	21	27	573	523	0.04379562
193679	9	18	162	284	0.060538117
441805	2	8	41	109	0.066666667
155557185	25	61	519	505	0.083984375
11556558	6	1	80	3	0.084337349
94320	40	21	421	265	0.088921283
155563897	18	60	186	628	0.095823096
9974201	23	34	223	242	0.122580645
155511476	95	111	827	774	0.128669582
10114	16	10	77	115	0.135416667
71773126	52	66	418	432	0.138823529
155552638	76	109	660	664	0.139728097
163184367	59	15	120	401	0.142034549
14077830	94	132	731	836	0.144224633
9983614	41	106	286	693	0.150153218
11003773	32	48	233	295	0.151515152
71451956	28	20	140	151	0.164948454
64945	66	51	413	263	0.173076923
155538646	148	116	659	744	0.188168211
102501232	31	35	145	196	0.193548387
73347309	68	93	279	552	0.193742479
9548703	70	130	576	454	0.194174757
14463159	56	67	371	260	0.194928685
91471	137	215	912	891	0.195230172
5380976	105	112	559	515	0.202048417
137796780	45	65	242	298	0.203703704
114850	179	99	720	622	0.207153502
163005195	58	68	190	404	0.212121212
92097	15	26	76	114	0.215789474

Supporting Table S2. Top 25 molecules selected through ligand-based 3D shape screening and ranked based on their average "ROCS_TanimotoCombo" values utilizing the two most active AbbVie molecules (PubChem CIDs: 155538646 and 155511476) used as 3D shape queries.

	ROCS Query (155511476)		ROCS Query (155538646)		Average ROCS_TanimotoCombo	Average ROCS_Rank
Ligand ID	TanimotoCombo	Rank	TanimotoCombo	Rank		
53266	1.003	1	0.822	5	0.9125	3
160487	0.944	6	0.827	4	0.8855	5
5320382	0.941	7	0.807	9	0.874	8
114829	0.96	2	0.788	23	0.874	12.5
5282073	0.946	4	0.788	22	0.867	13
632135	0.898	26	0.808	7	0.853	16.5
193679	0.904	20	0.794	17	0.849	18.5
46886723	0.924	11	0.78	29	0.852	20
115067	0.905	19	0.776	32	0.8405	25.5
5281601	0.905	18	0.773	35	0.839	26.5
5316844	0.914	13	0.765	45	0.8395	29
92775	0.924	10	0.764	49	0.844	29.5
5280443	0.899	24	0.765	46	0.832	35
96539	0.879	46	0.785	25	0.832	35.5
5281617	0.897	27	0.764	50	0.8305	38.5
5322078	0.883	40	0.77	39	0.8265	39.5
10450045	0.873	58	0.786	24	0.8295	41
5319081	0.892	30	0.758	63	0.825	46.5
198910	0.888	35	0.76	58	0.824	46.5
5320053	0.86	74	0.781	28	0.8205	51
11601633	0.905	17	0.75	86	0.8275	51.5
439246	0.911	15	0.749	89	0.83	52
5318980	0.859	77	0.78	30	0.8195	53.5
5281698	0.861	70	0.772	37	0.8165	53.5
72303	0.878	47	0.759	61	0.8185	54

Supporting Figure S4. The induced-fit docking modes of 8 known ApoE4 ligands reported by the AbbVie Pharmaceuticals [1]. The ligands are shown as gray, while the Tryptophan34 residue of ApoE4 protein (PDB ID: 8CE0) is shown as green.

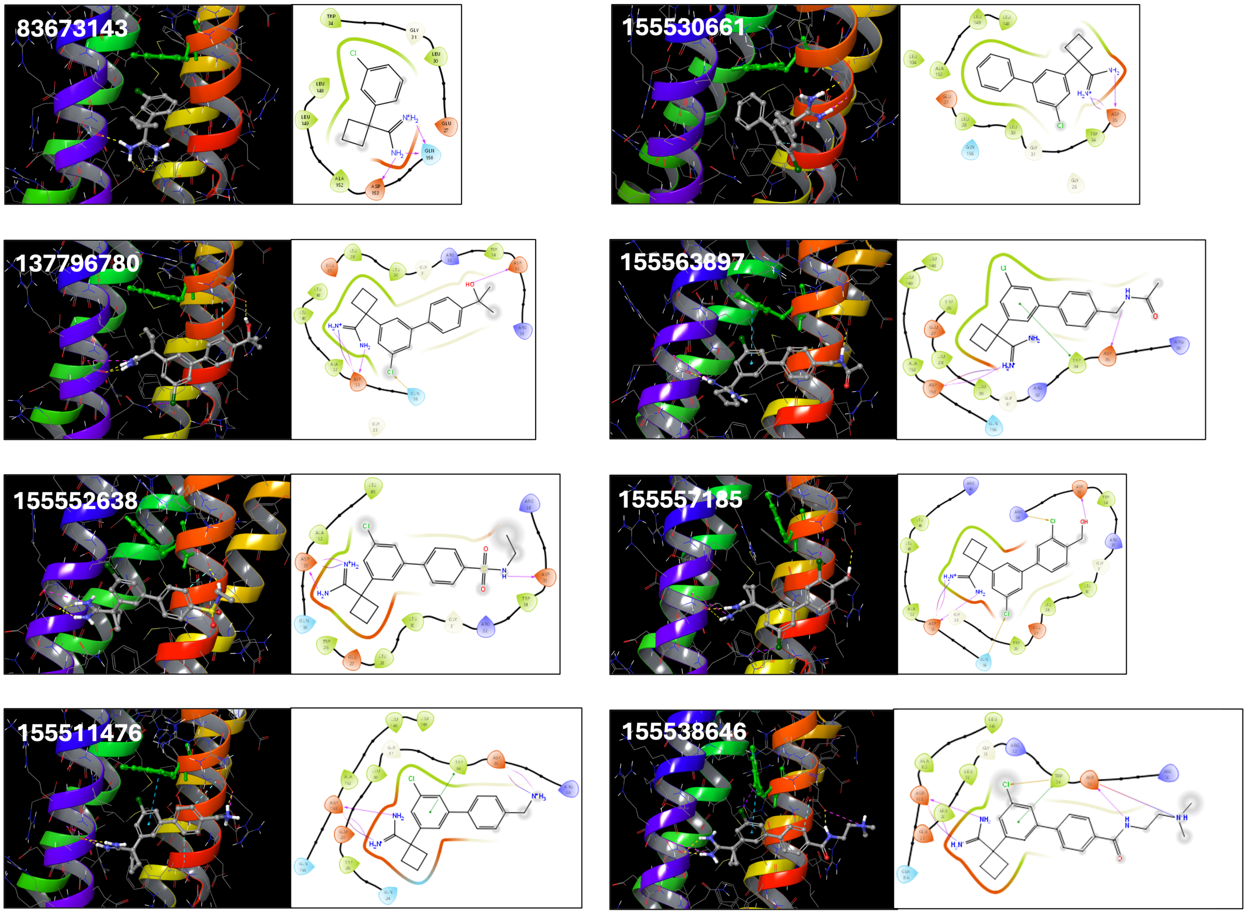

References:

1. Petros, A.M.; Korepanova, A.; Jakob, C.G.; Qiu, W.; Panchal, S.C.; Wang, J.; Dietrich, J.D.; Brewer, J.T.; Pohlki, F.; Kling, A.; Wilcox, K.; Lakics, V.; Bahnassawy, L.; Reinhardt, P.; Partha, S.K.; Bodelle, P.M.; Lake, M.; Charych, E.I.; Stoll, V.S.; Sun, C.; Mohler, E.G. Fragment-Based Discovery of an Apolipoprotein E4 (apoE4) Stabilizer. J Med Chem. **2019**, 62(8), 4120-4130. https://doi.org/10.1021/acs.jmedchem.9b00178
